# Friends and neighbours as non-kin caregivers of seriously ill patients at the end of life: a scoping review of experiences, individual capacities, support needs and services

**DOI:** 10.1186/s12904-025-01962-5

**Published:** 2025-12-11

**Authors:** Laura Stemme, Katharina Doll-Nikutta, Maria Heckel, Franziska A. Herbst

**Affiliations:** 1 Institute for General Practice and Palliative Care, Hannover Medical School, Carl-Neuberg-Strasse 1, Hannover, 30625 Germany; 2 Department of Prosthetic Dentistry and Biomedical Materials Science, Hannover Medical School, Hannover, Germany; 3 Department of Palliative Medicine, Friedrich Alexander University Erlangen Nuremberg Faculty of Medicine, University Hospital Erlangen, Erlangen, Germany

**Keywords:** Caregiver burden, Social support, Palliative care, Terminal care, Friends, Caregivers

## Abstract

**Background:**

The number of older individuals in need of care is projected to increase in the coming years. As support from family members is becoming increasingly limited, often due to geographical distance, non-kin caregivers, such as neighbours, friends, and acquaintances, are assuming an increasingly significant role in providing support. The present scoping review aimed to identify, describe and synthesise existing evidence on the experiences, individual capacities and support needs of informal non-kin caregivers of patients at the end of life, as well as the support services available to them.

**Methods:**

The review followed the methodological framework of Arksey and O’Malley. A search was conducted in four electronic databases (CINAHL, PsycInfo, PubMed, Web of Science Core Collection) on 10 October 2022 using a highly sensitive search strategy, and updated on 8 January 2025. Reference lists of the included studies were also hand-searched.

**Results:**

Two authors independently reviewed the titles and abstracts of 2,931 articles and screened 128 full texts. Seven articles met the inclusion criteria, and a further three articles were identified through hand searching. The analysis revealed seven overarching themes: (1) the duration and extent of non-kin caregiving, (2) caregiving relationships, (3) enabling care at home, (4) support wishes and needs, (5) modes of support, (6) non-kin caregivers as ‘hidden’ caregivers and (7) caregiving burden. Notable research gaps were identified, particularly regarding the low visibility of friends and neighbours in informal care, the lack of clear differentiation from family caregivers and the limited access to appropriate support and palliative care services.

**Conclusions:**

The findings showed that informal non-kin caregivers of seriously ill patients at the end of life remain under-recognised, despite the significant responsibilities they undertake. Future research is needed to improve access to tailored support and to distinguish their experiences from those of family caregivers.

**Supplementary Information:**

The online version contains supplementary material available at 10.1186/s12904-025-01962-5.

## Background

The proportion of older individuals living in single-person households is rising in many countries. In 2023, 31.6% of the population aged 65 and over in Europe were living alone [1], and similar figures (31% and 37%, respectively) have been recorded in the UK [2] and Austria [3]. According to the German Federal Statistical Office [1], older individuals are more than twice as likely to live alone compared to the general population. Older individuals account for 35% of all single-person households [4, 5]. This trend has intensified in recent years, driven by demographic shifts such as population ageing and changes in family structures [1, 4, 6, 7]. The care and support needs of older individuals living alone constitute a significant challenge for social and healthcare services [5].

In Germany, it is estimated that approximately five million individuals currently require care, with half between the ages of 75 and 89. The female-to-male ratio in this group is roughly 2:1. Approximately 800,000 individuals receive full inpatient care, while the majority (4.1 million) are cared for at home [8]. Of these, 2.5 million rely exclusively on relatives for care, and 1 million receive care with the assistance of professional services [8].

Pleschberger et al. [9] observed in 2015 that ‘there has been increased international attention on informal caregivers in palliative care in recent years but only a marginal number of studies have shown a specific interest in non-kin-carers’, and that research on non-kin caregiving relationships rarely addresses end-of-life situations [7]. To date, most studies involving non-kin caregivers have focused on the experiences and support needs of relatives – particularly family members – caring for patients at the end of life [10–16]. Marco et al. [11] identified key needs of family caregivers, such as emotional support, knowledge of the specific illness, and practical assistance. Similarly, Mitnick et al. [12] emphasised the critical role of family caregivers in providing routine and complex care, while also highlighting the associated physical, emotional, and financial burdens. Mehta et al. [13] demonstrated that family members almost invariably assume caregiving roles when supporting terminally ill patients. Broady et al. [10] emphasised the physical, emotional, psychological, and financial burdens of providing end-of-life care for a family member or friend with dementia. However, although this study encompasses friends as non-kin caregivers, their perspectives remain implicit and are not examined in depth. Furthermore, Gott *et al.’s* [17] *study* includes friends and neighbours as informal caregivers; however, the results do not distinguish between relatives and friends/neighbours as non-kin caregivers.

In practice, care is frequently provided by a wider support network that includes not only family members but also friends and other social contacts [13, 18–22]. The Alzheimer’s Association [19] underscores the pivotal role that close friends, alongside family members, play in the caregiving process for patients with dementia at the end of life. In a similar vein, Andershed et al. [20] emphasised the significance of informal non-kin caregivers’ involvement in caregiving, highlighting their capacity to provide support and actively participate in various aspects of caregiving. Reigada et al. [21] reported in their study that informal non-kin caregivers assume a range of tasks and help ensure continuity of caregiving for patients at the end of life.

It is therefore important to consider whether – and to what extent – individuals outside the family may assume comparable caregiving roles. The present scoping review explored the experiences, individual capacities and support needs of informal non-kin caregivers of seriously ill patients at the end of life, as well as the support services available to them.

In the present manuscript, the authors define the term ‘individual capacities’ as the personal, social, and material resources that are available to caregivers, enabling them to cope with and adapt to the demands of providing care. The term ‘support needs’ is defined as the emotional, informational, practical, and communicative requirements that arise during the process of caregiving and that informal caregivers of patients at the end of life face in order to manage their caregiving responsibilities and maintain their own wellbeing [11, 23].

### Motivations for the scoping review

The scoping review aimed at providing a comprehensive overview of the experiences, individual capacities and support needs of informal non-kin caregivers of seriously ill patients at the end of life, as well as the support services available to them, both nationally and internationally. Different from a systematic review, it did not assess the quality of evidence, but instead considered a broad range of studies, in order to minimise the risk of omitting relevant research. The review also sought to identify gaps in current knowledge, provide recommendations for further research and highlight care issues warranting further attention from social and health policy perspectives.

#### Study aim

The review aimed to address the following research questions:

1. What is currently known from the literature about the experiences, individual capacities and support needs of non-kin caregivers of seriously ill patients at the end of life?
2. What is currently known from the literature about the support services available to non-kin caregivers of seriously ill patients at the end of life?

## Design and methodology

### Scoping review steps

The review followed the five-step methodological framework of Arksey and O’Malley [24]: (1) identification of the research question(s), (2) identification of relevant studies, (3) selection of studies, (4) data extraction and charting and (5) summarisation and reporting of the results. The process followed the PRISMA-ScR checklist guidelines [25].

### Languages

The review included publications in both German and English. English-language publications were included, as English is the principle international language of science. Publications in German were also included because two of the authors are undertaking a research project on non-kin caregivers in end-of-life care (since July 2024) and sought to gain an overview of the literature from German-speaking countries.

### Inclusion and exclusion criteria

For the purposes of the study, a ‘non-kin caregiver’ was considered an individual providing care to a patient at the end of life with whom they had no familial relationship. Such caregivers included friends, acquaintances and neighbours. Non-kin caregivers who were organised within formal networks (e.g., hospice volunteers) were excluded from the research.

Research was included irrespective of the study design or publication type (e.g., anthology, monograph, original research article, published or unpublished thesis), in order to obtain the most comprehensive set of material for the review. Conference contributions and studies not published in full text were excluded. Review articles were considered in the discussion but excluded from the data analysis, which included only primary literature. The reference lists of review articles were screened, and any original research meeting the inclusion criteria was incorporated. To identify additional studies not retrieved through the database searches, reference lists of the included studies were hand searched.

The following inclusion and exclusion criteria were established: References were included if they focused on: (i) participants aged 18 years or older, (ii) informal non-kin caregivers, (iii) seriously ill patients at the end of life. The authors included (iv) anthologies, monographs, original article contributions, or (unpublished) theses, (v) in German or English language, (vi) when the paper was available. The exclusion criteria were defined as: (i) participants under 18 years, (ii) informal family caregivers or other family members, (iii) patients not at the end of life, (iv) conference abstracts or studies not published in full, and/or (v) publications in languages other than German or English, and/or (vi) papers not accessible in full.

### Databases

Four databases were searched to identify relevant literature across medicine, psychology, nursing science and related disciplines: CINAHL, PsycInfo, PubMed and Web of Science Core Collection. Searches were conducted across the selected databases from their inception up to 4 October 2022, in order to gain a comprehensive overview of the current evidence base. An updated search was undertaken across all selected databases on 8 January 2025. The final set of identified references was imported into EndNote, Version 20 (Clarivate, Philadelphia, USA), for management. No additional analysis software was utilised in the subsequent process. Reference lists of relevant publications and review articles were hand searched to minimise the risk of omitting important literature. A follow-up search was conducted on 8 January 2025.

### Search strategy

An iterative literature search was conducted to maximise the retrieval of potentially relevant studies. The specificity and sensitivity of the search strategy were assessed through a preliminary search, which tested whether the proposed search terms could identify a key article already known to the authors [26] on informal non-kin caregivers of individuals at the end-of-life. Based on this, the search strategy was refined and adapted for each database. The final search strategy (see online supplemental file 1) included the terms ‘informal non-kin caregiver/s’, ‘end-of-life’, ‘experiences’, ‘support need/s’, ‘individual capacities’ and ‘support service/s’, together with their synonyms and related concepts, linked using the Boolean operator OR. To enhance retrieval, database-specific subject headings were applied, including CINAHL major and minor headings, PsycInfo major subject headings and PubMed Medical Subject Headings (MeSH). The Boolean operator NOT was used to exclude non-relevant studies, including those focused on family caregivers or caregivers of terminally ill children.

### Analysis

The full texts were screened, and relevant information was extracted and compiled in tabular form. The data was categorised into four subgroups: (1) experiences, (2) support needs, (3) individual capacities, and (4) support services for informal non-kin caregivers of patients at the end of life. The utilisation of colour coding facilitated the identification of overlapping content and thematic similarities, thereby informing the subsequent development of key themes.

## Results

### Study selection

**Fig. 1:**
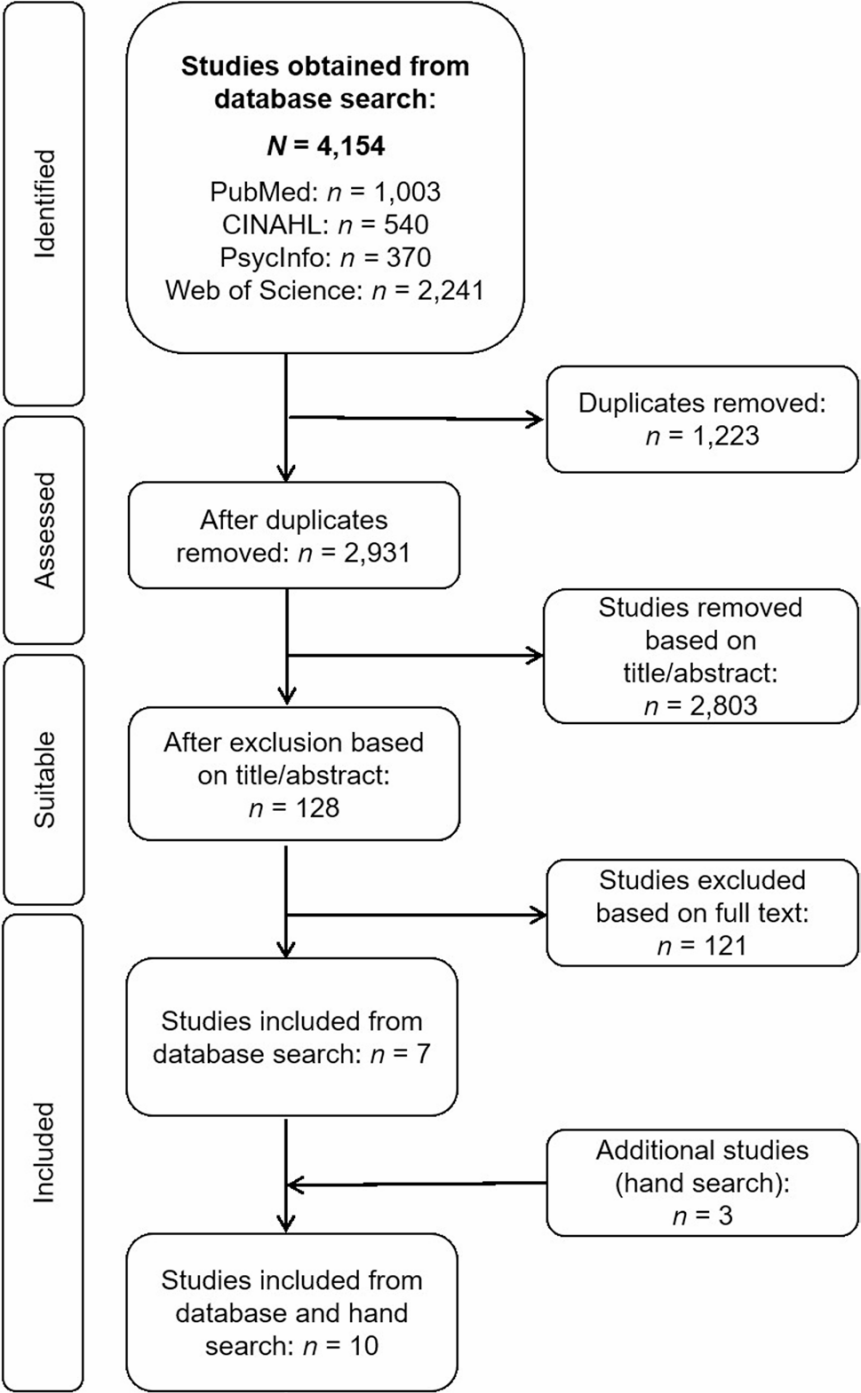
Study selection flow chart

The database search yielded 2,931 references after the removal of duplicates (see Fig. 1 for the study selection flow chart). In the first screening stage, two authors (LS and FAH/KDN) independently reviewed the titles and abstracts to assess relevance for inclusion. Abstracts were excluded if they focused on: (i) non-kin caregivers under 18 years of age, (ii) non-kin caregivers of patients under 18 years of age and/or (iii) non-kin caregivers of patients who were not seriously ill or not at the end of life. The main reasons for exclusion were documented and used to reach consensus. Based on this step, 2,803 studies were excluded. In the second step, the same authors evaluated the full texts of the remaining 128 studies against the inclusion criteria. This process identified seven studies [26–32] for inclusion. Reasons for exclusion at this stage included: (i) population not comprising friends, neighbours or acquaintances in non-kin caregiver roles (*n* = 101), (ii) care context not related to end of life (*n* = 3), (iii) publication type not meeting the criteria (e.g., not an original research article; *n* = 13) and (iv) full text not available (*n* = 2). A further three studies [33–35] were identified through hand searching. Two of these [33, 34] were identified within a review article [36] that had been excluded during screening.

### Characteristics of the included studies

The included studies varied in their geographical location and research focus (see Table 1 for details). The majority (*n* = 4) were conducted in Australia [27, 29, 33, 34], followed by the UK (*n* = 3) [28, 31, 32], Austria (*n* = 2) [26, 35] and Belgium (*n* = 1) [30]. The studies were published between 1998 and 2024, with four appearing between 2023 and 2024 [30–32, 35] and five between 2011 and 2020 [26, 27, 29, 33, 34]. Qualitative methods were used in six studies [26–29, 32, 35], quantitative methods in three [30, 33, 34], and mixed methods in one [31]. Five of the qualitative studies [26, 27, 29, 32, 35] and all three quantitative studies [30, 33, 34] were published as original research articles, while one qualitative study [28] was based on secondary data analysis. Sample sizes for the qualitative studies ranged from 6 to 89 non-kin caregivers, yielding a total of 145 participants across five studies [26–28, 31, 32, 35]. One qualitative study [29] examined 35 care networks that included caregiving friends, but did not specify the exact number of non-kin caregivers. The three quantitative studies included a combined total of 982 participants, with individual sample sizes ranging from 228 to 380 [30, 33, 34].

**Table 1:** Summary of the included studies

Author(s) (year); no. in references	Publication type	Geographic location of study (non-kin caregiver)	Research aim	Study design	Study population (non-kin caregivers) and sample size	Results regarding (1) experiences, (2) support needs, (3) individual capacities and (4) support services of informal non-kin carers of patients at the end of life
Young et al. (1998) [28]	Secondary data analysis (qualitative)	UK	Investigate the role of friends and neighbours as caregivers for patients at the end of life, and examine their relationships with families and/or professionals.	Secondary data analysis of the qualitative, retrospective survey by Cartwright and Seale [37], with a primary focus on services provided to the patient at the end of life. The analysis included semi-structured interviews with self-identified ‘close friends’ or ‘friends/neighbours’, focusing on services received by bereaved caregivers. Descriptive qualitative analysis was applied.	Secondary analysis of Cartwright and Seale’s study, based on semi-structured interviews with 89 friends and neighbours who cared for a deceased person at the end of life (*n* = 7 close friends, *n* = 39 friends, *n* = 43 neighbours).	(1) Friends described their relationships with the deceased in terms of kinship, particularly to underscore the closeness of the bond. (1) 63% (*n* = 56) of caregiving friends and neighbours had known the patient for more than 20 years. (1) Friends and neighbours were involved in intimate and demanding caregiving tasks, including personal hygiene and bodily care, housekeeping and pet care for the patient at the end of life. (2) Friends and neighbours reported that they could have benefited from more help with these tasks. (4) Specialist professional caregivers employed to provide support did not assume all necessary responsibilities and therefore did not offer complete relief. (1) Close caregiving friends and neighbours experienced these caregiving tasks as burdensome. (1) Caregiving friends and neighbours felt a strong sense of duty and obligation towards the patient. (1) They also reported not receiving detailed information about the patient’s condition from doctors or specialist care professionals, due to their status as non-family members. (1) Only in rare cases did professionals consult caregiving friends as sources of assistance. (1) Some friends reported feeling excluded or disenfranchised in the informal caregiving process by professionals and/or family members.
Burns et al. (2011) [33]	Original research article (quantitative)	Australia	Explore the role of friends as caregivers of patients at end of life.	Quantitative study design: survey data collected through the South Australian Health Omnibus Survey (HOS) from 2001 to 2007, including questions on patient and caregiver characteristics [38, 39]; data were analysed using descriptive statistics.	Total respondent population of *n =* 23,588, including *n =* 380 friends who acted as caregivers for a patient at the end of life who ultimately died from a life-limiting illness. Of these, 265 were actively engaged in caregiving tasks, comprising 65 day-to-day ‘hands-on’ caregivers and 200 intermittent ‘hands-on’ caregivers.	(1) 29.7% (*n* = 54) of all actively caregiving friends had been involved in caregiving for at least 12 months, while 49.5% (*n* = 90) had provided care for up to 6 months, and a further 28.6% (*n* = 52) had provided care for 3 months or less. (1) Only a small percentage of actively caregiving friends (3.4%, *n* = 8) were unsure about what to expect from caregiving; 47.5% (*n* = 113) stated that the period from their friend’s diagnosis until their friend’s death was (much) worse than anticipated. (1) 78.6% (*n* = 44) of all actively caregiving friends expressed a willingness to take on caregiving responsibilities again. (1) When care was provided by a friend, it was more likely that the deceased died at home (32%) and that specialised palliative care services were involved (70%). (2) 35.8% (*n* = 38) of actively engaged caregivers sought help – or wished they had sought help – to cope with grief. (3) 89.2% (*n* = 74) of caregiving friends did not experience significant financial strain. None gave up their jobs, three reduced their working hours and one utilised other leave entitlements.
Lewis et al. (2014) [27]	Original research article (qualitative)	Australia	Examine the nature of social capital among patients at the end of life and informal caregivers within a lower socioeconomic population.	Qualitative study design: semi-structured interviews conducted using a Social Capital Questionnaire (SCQ) comprising 16 items developed by the researchers, focusing on individual, community, and civil society networks and relationships. Reporting was carried out in accordance with the COREQ [40] criteria.	Patients at the end of life (*n* = 16), including two who were cared for by a neighbour, and informal caregivers (*n* = 6) with social and economic needs, all from a lower socioeconomic area (as self-reported).	(1) Level of caregiving support provided by neighbours depended on patients’ needs. (1) Neighbourly support was both practical and emotional – for example, assistance in the household, with everyday tasks, transportation, and even assuming Power of Attorney for the person at the end of life.
Burns et al. (2015) [34]	Original research article (quantitative)	Australia	Report the differences between patients at the end of life receiving care in rural versus urban settings and the differences in the patient and caregiver population itself.	Quantitative study design: data collected through the South Australian Health Omnibus Survey (HOS) from 2001 to 2007; data were analysed using descriptive statistics to summarise the characteristics of respondents and their answers.	‘Hands-on’ caregivers (*n* = 2,027), including caregiving friends in urban (*n* = 244) and rural (*n* = 130) settings.	(1) The proportion of rural friends (20.2%, *n* = 130) acting as ‘hands-on’ caregivers for patients at the end of life was greater than that of urban friends (17.7%, *n* = 244). (1) 7.6% (*n* = 33) of urban friends provided daily ‘hands-on’ care, compared to 13.2% (*n* = 33) in rural areas. (1) 21.2% (*n* = 124) of urban friends provided intermittent ‘hands-on’ care, compared to 28.8% (*n* = 81) of rural friends. (1) 23.9% (*n* = 92) of urban friends provided rare ‘hands-on’ care, compared to 16.1% (*n* = 25) of rural friends.
Pleschberger and Wosko (2017) [26]	Original research article (qualitative)	Austria	Better understand the involvement of non-kin caregivers of older patients living alone, and gain insight into the challenges they face at the end of life.	Qualitative study design: exploratory, retrospective in-depth interviews conducted using an interview guide comprising 12 open-ended questions developed by the researchers. The interviews were coded systematically and the content was subsequently summarised into broader thematic categories.	Non-kin caregivers (*n* = 15), comprising friends (*n* = 8) and neighbours (*n* = 7), as well as professional caregivers (*n* = 8).	(1) Caregivers observed an increasing need for support due to declining health and growing physical care needs. (1) The main challenge reported by non-kin caregivers was the burden of advancing physical care needs, coupled with anxieties when confronted with death and dying. The data indicate a heightened risk of overburdening neighbour caregivers, particularly in the final phase of life. (1) Patients who died at home with the support of non-kin caregivers also received care from professional palliative providers. (1) Some differences between friends and neighbours in caregiving were noted: involvement with friends often had no clear beginning and typically evolved gradually, often starting with a small act of help. Friends appeared more willing than neighbours to take on physical care tasks. (1) Friends and neighbours were motivated to care for various reasons – including intrinsic motivation, material reciprocity and emotional reciprocity. Friends’ motives were more often rooted in compassion than affection. (1) Caregivers were present for patients multiple times a day (e.g., through visits or phone calls). (1) Caregiver engagement often lasted several years, and no caregivers considered giving up caregiving during the end-of-life process. (1) In some cases, older care recipients became part of the neighbour’s family life. However, some caregivers experienced conflict within their own families due to the significant time invested in caregiving. (2) Caregivers – and particularly those without prior caregiving experience – navigated the final phase of the patient’s life more easily when supported and informed by professional care services. (3) Caregivers with prior experience in caregiving benefited greatly from it. (4) Caregivers were supported by specialised palliative care during their involvement, including advance information and 24-hour availability.
Leonard et al. (2020) [29]	Original research article (qualitative)	Australia	Investigate the relationship between informal caregivers (and their networks) and formal service providers, and analyse and optimise caregiving networks.	Qualitative study design: third-generation social network analysis, based on individual carer interviews, focus groups with caring networks and outer network interviews. Qualitative content analysis was employed.	Networks (*n* = 36) of individuals who cared for a now-deceased person, of which 35 included friends as non-kin caregivers. The deceased had died within the past 3 years due to a life-limiting illness.	(1) One caregiving friend had moved to the area and increased his use of formal service provision, as he had previously had few acquaintances to support him. (1) Another caregiving friend had become a formal service provider, which had increased the involvement of other formal service providers within the network and formed a stronger bond between them.
D’Eer et al. (2023) [30]	Original research article (quantitative)	Belgium	Explore neighbourhood participation in serious illness, death and loss, and examine how social cohesion influences citizen engagement in these critical life events.	Quantitative study design: a cross-sectional survey was conducted from February to April 2021 using a questionnaire comprising five main concepts. Data were entered into LimeSurvey and analysed using SPSS statistical software.	*N* = 704 fully completed the questionnaire, all aged 18 or over and randomly selected by a city official, including neighbours as non-kin caregivers (*n* = 228). The survey was conducted in two neighbourhoods within suburban municipalities.	(1) A total of 32.4% (*n* = 228) of participants reported providing assistance to a close neighbour who was seriously ill or in need of help. (1) Individuals were more inclined to support close neighbours than to engage in broader neighbourhood activities related to ‘serious illness, death and loss’, particularly in the absence of a personal connection to the individual concerned.
Mogan (2023) [31]	Original research thesis (mixed methods)	UK	Examine the experiences of, and available support services for informal caregivers of patients with dementia living at home at the end of life; understand how best to support these caregivers; and analyse the role of ‘compassionate community’ initiatives in providing end-of-life care at home for patients with dementia and their caregivers.	Mixed-methods study design: semi-structured face-to-face interviews and quantitative cross-sectional questionnaires (covering three main topics and 10 questions) were conducted within a convergent parallel design. Data were analysed using six-step thematic analysis (qualitative) and descriptive statistics (quantitative).	Informal caregivers (*n* = 29), including two friends, who provided end-of-life care at home for a person with dementia. Questionnaire: ‘Compassionate Community’ projects (*n* = 24) supporting patients with dementia and their caregivers.	(1) Participating friends identified pain management as a central component of end-of-life caregiving at home and highlighted the challenge of verbal communication with patients suffering from dementia. (1) Participating friends reported uncertainty regarding the dying process of patients with dementia and found it difficult to determine when the terminal phase had begun. (1) Participating friends felt insecure in emergency situations because they did not know whom to contact. During a crisis, they typically sought support from professional services. (1) Participating friends sought assistance from domiciliary care workers during the terminal phase of life for patients with dementia, particularly with personal care tasks such as washing, dressing and managing incontinence. (1) Neighbours provided support through transportation, gardening, pet care, housekeeping and cleaning. (1) Caregiving neighbours regularly visited patients with dementia at the end of life, typically for one hour per week
Patel et al. (2023) [32]	Original research article (qualitative)	UK	Analyse the implementation of compassionate communities in home-based end-of-life care, explore the experiences gained and assess whether such communities represent a form of therapeutic landscape.	Qualitative study design: semi-structured interviews, focus groups and diary logs were collected between 2016 and 2018 across two hospices and one community organisation. Braun and Clarke’s [41] thematic analysis served as the framework for subsequent interpretative analysis, with data management conducted using NVivo 10.	Total respondent population of *n* = 57, including one friend acting as a caregiver for a person over the age of 50 at the end of life, with a prognosis of at least 6 months, living at home.	(1) Friends and neighbours, as informal caregivers, were represented in both the inner and outer circles of care. The inner circle comprised participants to whom caregivers provided support on a daily or weekly basis, while the outer circle comprised those with whom interactions were less frequent. (1) A wide network of caregiving friends enabled the sharing of caregiving responsibilities. (1) Support from caregiving friends was provided in practical ways and through companionship, social interaction and assistance at home. (1) Patients at the end of life valued their relationships and social interactions with caregiving friends, as well as with other formal and informal caregivers.
Wosko et al. (2024) [35]	Original research article (qualitative)	Austria	Identify the characteristics of support arrangements for older patients living alone at home, and explore the involvement and potential of non-kin caregivers within these arrangements.	Qualitative study design: semi-structured face-to-face interviews were conducted and analysed using a coding strategy based on Corbin and Strauss’s grounded theory [42], resulting in the development of sociograms for each case.	Older individuals (aged 67 to 99) (*n* = 32) experiencing a progressive decline due to illness or frailty, without cognitive impairment. All were supported by at least one non-kin caregiver (friends, neighbours, or acquaintances) and had no relatives living nearby.	(1) Friends represented a significant proportion of the support network for older individuals living alone (no explicit quantitative data available). (1) Friends and neighbours provided a broad spectrum of support, encompassing both instrumental and emotional assistance. Instrumental support included help with household tasks, transportation and driving, and shopping. (1) Neighbours were involved in a more diverse range of supportive activities than friends and acquaintances. In addition to the services mentioned above, neighbours supported older individuals living alone by caring for pets, cooking, gardening and performing manual tasks (particularly provided by male neighbours). Female neighbours offered support with organisational matters. (1) Personal care was primarily provided by female neighbours, not by friends. (1) Financial management – including holding power of attorney – was carried out by friends, but not neighbours. (1) Support services were tailored to the unique needs of older patients and the capacities of individual friends to provide support. (1) A considerable degree of mutual trust was evident between supporting friends and patients.

### Findings of the included studies: descriptive analysis of topics

Seven key themes were identified inductively from the results of the included studies, relating to the experiences, individual capacities, support needs and support services available to informal non-kin caregivers of patients at the end of life: (1) the duration and extent of non-kin caregiving, (2) caregiving relationships, (3) enabling care at home, (4) support wishes and needs, (5) modes of support, (6) non-kin caregivers as ‘hidden’ caregivers and (7) caregiving burden.

#### Duration and extent of non-kin caregiving

Six studies addressed the duration and extent of non-kin caregiving of patients at the end of life [26, 27, 31–33, 35]. Two studies reported that caregiving was provided over a period of several months [26, 33], while five studies described the extent of care delivered by non-kin caregivers [26, 27, 31, 32, 35]. Burns [33] found that 28.6% (*n* = 52) of caregiving friends provided care for up to 3 months, 20.9% (*n* = 38) for 4 to 6 months, 6.0% (*n* = 11) for 7 to 9 months, and 14.8% (*n* = 27) for 10 to 12 months. The largest group (29.7%, *n* = 54) provided care for more than 12 months. Similarly, Pleschberger et al. [26] reported that many caregiving relationships extended over several years, with some lasting more than 15 years. Notably, none of the non-kin caregivers in their study ended the relationship during the patient’s end-of-life phase, even as health deteriorated.

The studies also revealed differences between friends and neighbours in the development of caregiving relationships [26]. Among friends, the transition from a social to a caregiving role tended to be fluid, without a clearly defined starting point. In contrast, caregiving by neighbours often began with small, practical acts of help (e.g., giving a lift to the supermarket) that gradually expanded over time. One neighbour in Pleschberger et al.’s [26] study recalled that the relationship began with a one-time act, such as bringing groceries or watering plants, and evolved into ongoing support over several years. Caregiver motivations also differed. For neighbours, Pleschberger et al. [26] identified compassion and a sense of social obligation as primary drivers. One caregiving neighbour described feeling sorry for the patient, noting that she was alone and looked forward to regular visits. The authors concluded that there is ‘a difference in the nature of the relationship [of neighbours] compared to friends‘ [26], though without elaborating further.

Beyond this difference between caregiving friends and neighbours, the literature also highlights variations between urban- and rural-dwelling friends. Burns et al. [34], in a quantitative study, found that a significantly higher proportion of rural friends (20.2%, *n* = 131) described themselves as ‘hands-on’ caregivers for patients at the end of life compared to urban friends (17.7%, *n* = 244). Similarly, 13.2% (*n* = 33) of rural friends provided ‘daily hands-on’ care, compared to 7.6% (*n* = 33) of urban friends. Conversely, a smaller proportion of rural friends (16.1%, *n* = 25) provided ‘rare hands-on’ care compared to their urban counterparts (23.9%, *n* = 92).

With respect to the extent of caregiving, Lewis et al. [27] reported two cases in which patients indicated that support from neighbours varied according to their needs, increasing when those needs intensified. Similar findings were observed by Pleschberger et al. [26], who noted that non-kin caregivers became more involved as patients’ health declined. This often meant being available several times a day – including early mornings, late evenings and during the night. One caregiving friend in Pleschberger et al.’s [26] qualitative study described a variable daily pattern of up to three visits and as many as three phone calls. Patel et al. [32] described the extent of support from friends and neighbours as occurring on a ‘daily or weekly basis’ or ‘less regularly’. In contrast, Mogan’s [31] study found that caregiving neighbours typically provided approximately one hour of support on a weekly basis.

#### Caregiving relationships

Six of the included articles addressed caregiving relationships [26, 28, 30, 32, 34, 35]. While five focused on the relationship between caregivers and patients at the end of life [28, 30, 32, 34, 35], Pleschberger et al. [26] examined relationships between caregivers and their own relatives.

The literature indicates that caregiver–patient relationships at the end of life vary widely. Four studies reported the ages of informal non-kin caregivers of seriously ill patients [26, 30, 31, 33], with three finding similar mean ages: 62.8 years [26], 63.2 years [30] and 63.5 years [31]. By contrast, Burns et al. [33] reported a lower mean age of 52.3 years for caregiving friends, with daily hands-on caregivers averaging 57 years and intermittent hands-on caregivers averaging 50.7 years.

Some caregiver–patient dyads had relatively formal relationships, but most described a close and meaningful bond. One neighbour in Pleschberger et al.’s [26] qualitative study emphasised that the patient was not part of their family, yet the patient was nonetheless integrated into the caregiver’s family life. Similarly, Young et al. [28] found that 63% (*n* = 56) of caregiving neighbours and friends had known the patient for over two decades prior to their death. Many participants in Young et al.’s work expressed the depth of these bonds by using the term ‘kinship’. One neighbour likened his relationship with the patient to that of a father, while two other participants described the patient as being ‘like a sister’ to them. Wosko et al. [35] also noted the presence of strong mutual trust between patients and their caregiving friends, illustrated by the fact that caregivers often held keys to patients’ homes.

While the studies above examined relationships between caregivers and patients at the end of life, Pleschberger et al. [26] investigated the ways in which caregiving affected relationships within caregivers’ own families. The study identified instances of conflict, with some non-kin caregivers reporting tensions arising from the time and emotional commitment required. One participant recounted that her husband had criticised her for being too engaged and involved with the non-kin patient.

#### Enabling care at home

Four studies reported on the role of non-kin caregivers in enabling end-of-life care at home [26, 29, 31, 33]. Three [26, 29, 33] indicated that, for some patients, the possibility of dying at home was highly valued. Burns et al. [33] found that when a friend provided care, the likelihood of a patient dying at home increased to 32%. In these cases, non-kin caregivers were involved alongside other carers in home-based care, sometimes undertaking physical care tasks. Specialised palliative care services were involved in 70% of such cases [33]. Similarly, Pleschberger et al. [26] found that patients who died at home with support from non-kin caregivers also received care from professional palliative teams. Mogan [31] reported comparable findings, noting that domiciliary care workers supported caregiving friends of patients with dementia at the end of life by assisting with personal care tasks.

#### Support wishes and needs

Two studies addressed the specific support wishes and needs of non-kin caregivers [28, 33], though information was limited. In Young’s [28] qualitative study, non-kin caregivers expressed a general desire for greater assistance with caregiving tasks. Burns et al. [33] reported that 35.8% (*n* = 38) of caregivers actively sought – or wished for – support during their bereavement.

#### Modes of support

Six studies examined the broad spectrum of support provided by non-kin caregivers to patients at the end of life [26–28, 31, 32, 35]. Five reported on practical support [27, 28, 31, 32, 35], and four on emotional support [26, 27, 32, 35].

Practical assistance included the provision of transport for medical appointments [27, 31, 35] and tasks such as meal preparation, management of administrative matters, bill-paying (e.g., utilities, rent) and running errands such as buying clothes [27, 35]. Non-kin caregivers also looked after patients’ pets, maintained the household (including cleaning) and gardened [28, 31, 35]. Pain management was noted as a practical task in Mogan’s [31] study.

Three studies reported that friends and neighbours also undertook intimate caregiving tasks related to personal care and hygiene [26, 28, 35]. For example, one friend in Young’s [28] study described changing a patient’s clothes three times a day. Pleschberger et al. [26] observed that friends were generally more willing than neighbours to perform physical end-of-life care, whereas Wosko et al. [35] found that personal care was provided exclusively by neighbours, who offered a broader range of support than caregiving friends.

Emotional support, as described by Patel et al. [32], included ‘keeping company’ and engaging in social interaction. Some non-kin caregivers also assumed significant legal and decision-making responsibilities. Lewis et al. [27] described a case in which a non-kin caregiver held power of attorney for medical decisions, while Wosko et al. [35] reported cases in which caregiving friends were given financial power of attorney.

#### Non-kin caregivers as ‘hidden’ caregivers

One study examined the position of non-kin caregivers within the broader caregiving framework for patients at the end of life [28]. In a qualitative secondary data analysis, Young et al. [28] explored the relationships between non-kin caregivers and family members and health professionals, finding that non-kin caregivers in their sample often felt disenfranchised and excluded by these parties. Caregiving friends reported that specialist palliative care providers and doctors often withheld detailed information about the patient’s health status, and one participant (it is unclear whether this was a friend or neighbour) stated that they would have liked to know everything about the patient’s condition in order to provide more effective support. Another participant recounted taking the patient to medical appointments but always waiting outside, citing their non-family status and respect for the patient’s privacy. Professional caregivers were reported to approach caregiving friends for assistance only rarely.

#### Caregiving burden

Four studies addressed the caregiving burden experienced by non-kin caregivers of patients at the end of life [26, 28, 31, 33]. Reported burdens included emotional strain from confronting dying and death, the demands of care provision, financial pressures and impacts on employment. All four studies described negative consequences for caregiving neighbours and friends. More specifically, Mogan’s [31] mixed-methods study found that non-kin caregivers often felt insecure when faced with the dying process and related emergencies. One female caregiving friend described the patient’s final week as a ‘battle’. Pleschberger et al. [26] similarly noted the emergence of anxieties when caregivers were confronted with death, with one male participant describing himself as ‘overburdened’ and unsure how to manage the process. Burns et al. [33] reported that only a small proportion (3.4%, *n* = 8) of caregiving friends lacked understanding of what caregiving would involve. However, nearly half (47.5%, *n* = 113) felt that the period from illness onset to death was significantly worse than expected. Young’s [28] findings align with this, documenting substantial mental burdens. Participants frequently expressed a strong sense of duty, with one noting that they ‘could not zoom off’ and needed to ensure the patient’s wellbeing. Both Young [28] and Pleschberger et al. [26] identified caregiving tasks as a source of strain. In Young’s study, one caregiving friend admitted feeling relief when the burden ended with the patient’s death. Pleschberger et al. also found that prior caregiving experience was perceived as advantageous in managing end-of-life situations.

In contrast, Burns’s [33] quantitative study found that 89.2% (*n* = 74) of caregiving friends reported no financial burden, and only 3.6% (*n* = 3) had to use savings. Employment impacts were also minimal: none of the 261 participants gave up their jobs, only 3.6% (*n* = 3) reduced their working hours, and 1.2% (*n* = 1) took leave.

## Discussion

The present findings illustrate the experiences, individual capacities and support needs of informal non-kin caregivers of seriously ill patients at the end of life, as well as the support services available to them. Relationships between non-kin caregivers and friends, neighbours and acquaintances often last for several months or even years, in some cases continuing until the care recipient’s death. Caregiving intensity typically increases as the patient’s health deteriorates. The nature of these relationships can range from relatively formal to highly personal. Moreover, non-kin caregivers provide a wide range of support and play an important role in enabling patients to die at home, often working alongside professional palliative care services. Despite this, they may feel excluded by both the patient’s family and health professionals. Frequent burdens include emotional strain and caregiving-related stress, while financial impacts are generally limited. These findings align with those of Barker et al. [43], who noted that non-kin caregivers, similar to their family caregiving counterparts, often face substantial physical, psychological and social challenges when supporting dependent older adults.

Caregiving for a patient at the end of life is widely regarded as highly stressful, with both family and non-kin informal caregivers reporting feelings of anxiety [44]. The closer the relationship, the more profound the emotional impact is likely to be [45]. The reviewed studies also indicate that non-kin caregivers desire increased assistance, particularly in the patient’s final days and during the grieving process. This is consistent with prior research showing that end-of-life caregiving presents distinct challenges, and informal caregivers have specific needs. Both family and non-kin caregivers therefore require emotional and psychological support [18, 46–48] throughout the illness trajectory, to prepare them for loss and grief and to ensure access to bereavement support [49–51]. Furthermore, some family and non-kin caregivers may avoid seeking help due to a lack of awareness about how to access it [47, 48]. Further hesitation may also stem from non-kin caregivers’ reluctance to consider themselves ‘proper’ caregivers [43]. The similarity between the experiences of non-kin caregivers in this review and those of family caregivers reported in previous studies suggests that these groups may share comparable support needs.

In recognition of the caregiving roles already undertaken by non-kin caregivers, structured initiatives such as the ‘compassionate community’ have been developed to formalise and expand community-based end-of-life support. The compassionate community represents a public health model in palliative care emphasising shared societal responsibility for supporting patients at the end of life, alongside their caregivers. The term was first introduced by Kellehear [52], who argued that this model is an essential public health strategy [53] for fostering new and constructive ways of interacting, thereby building community capacity for end-of-life care [52, 54, 55]. Kellehear [53] calls upon all members of the public to assume responsibility, framing the care of patients in the terminal phase of illness as a collective obligation. Key objectives of the compassionate community include education, reduced stigma and active participation. Conversations about death are normalised and holistic care is provided that complements professional health services [53, 54]. Burns et al. [33] found that only a minority of non-kin caregivers sought assistance, consistent with Grindrod et al. [56], who suggested that reluctance to seek and accept external support may hinder the establishment of a compassionate community.

### Implications for practice

One support service for non-kin caregivers is the Healthy End of Life Project (HELP) [56], which provides evidence-based resources and a practical framework to assist informal caregivers in delivering end-of-life care at home and within community settings. Moreover, the ‘last aid course’, described by Bollig et al. [57], offers basic palliative care education to the general public and aims to encourage open discussion about death and dying. Both initiatives enhance knowledge and support structures for non-professional caregivers while improving the accessibility and effectiveness of palliative care services.

The last aid course, the HELP project and similar approaches [58–60] can serve as a foundation [57] and provide a framework for the development of compassionate communities [56]. Future efforts should focus on expanding existing support services and ensuring their accessibility. Many authors suggest that the most effective strategy for future generations will be a combination of educational initiatives aimed at increasing public understanding of palliative care and the development of compassionate communities [53, 55, 57, 61]. The presence of such communities may enhance the fulfilment of patients’ wishes—particularly the wish to die at home [57, 62].

### Implications for policy

The reviewed studies demonstrate that non-kin caregivers of seriously ill patients at the end of life provide extensive care. Despite their significant contribution, they are often regarded as ‘hidden caregivers’ and remain largely invisible within health and social care systems. It is therefore essential that non-kin caregivers be formally recognised at both local and national levels. In particular, legal definitions and targeted policies should ensure that their contributions are acknowledged and they are provided with appropriate support.

The findings also indicate that friends and neighbours acting as non-kin caregivers are sometimes excluded by both family members and healthcare professionals, and that such exclusion may negatively affect the quality of care provided. Policy measures should facilitate the integration of non-kin caregivers into professional care teams, such as by actively encouraging their involvement in care processes [63, 64] and/or granting them legal access to relevant patient information.

Although previous research [26] has not clarified whether non-kin caregivers incur direct costs such as parking fees, meal costs or telephone expenses, the present review suggests that financial burdens are generally not a major concern. Nevertheless, additional resources could help to sustain their commitment and ensure the long-term provision of care.

### Implications for future research

With regard to the disparities between previous research [26] and the present review concerning financial burdens, further research is necessary to determine the extent to which non-kin caregivers experience financial strain as a result of their role.

Non-kin caregivers, as an insufficiently researched group of caregivers, report a lack of fulfilment of their support needs. Further research should focus on improving access to these support services and expanding knowledge about caregiving of patients at the end of life.

### Limitations

The language-based search criterion may have led to the omission of relevant studies published in languages other than English or German. Grey literature, reports, conference proceedings, studies not published in full and unpublished works were also excluded. The search was limited to four databases, supplemented by a hand search of reference lists from selected articles. Relevant studies indexed in other databases, or absent from the reference lists reviewed, may therefore have been overlooked. A considerable number of references were excluded due to their ambiguous distinction between non-kin caregivers and other caregiver groups, making it difficult to determine the applicability of the findings to non-kin caregivers. This may have led to the exclusion of relevant literature.

With regard to the key themes ‘support wishes and needs’ and ‘non-kin caregivers as ‘hidden’ caregivers’, evidence was available from only two/one reference(s), respectively. Consequently, the current literature available does not allow to assert the generalizability of these results to non-kin caregivers in different contexts or scenarios.

Finally, the quality of the included evidence was not assessed, as the aim of this scoping review was to provide a broad overview of the literature rather than a critical appraisal. Given the urgent need for evidence to inform an ongoing research project, no formal quality assessment was undertaken. The authors acknowledge this as a methodological limitation.

## Conclusions

The present scoping review aimed to identify, describe and synthesise the available evidence on the experiences, individual capacities and support needs of informal non-kin caregivers of seriously ill patients at the end of life, as well as the support services available to them. The findings indicate that non-kin caregivers remain a largely invisible group, often regarding their involvement as a private matter and, in some cases, not even identifying themselves as caregivers. Nevertheless, they play a vital role in end-of-life care, frequently assuming significant responsibilities and providing extensive, meaningful support. Although a range of support services for caregivers exists, most non-kin caregivers express a need for additional assistance. Future research should focus on improving access to such services, enhancing knowledge of palliative care and building relevant caregiving skills. Non-kin caregivers are still under-researched, with most studies identified in this review originating from Australia, followed by the UK. Only three studies included participants from European countries, and none from Germany. Further research is therefore required in Germany and other European contexts.

Several research gaps were identified. Notably, only three quantitative studies and one mixed-methods study met the inclusion criteria, suggesting that qualitative approaches currently dominate the field and may be particularly well suited to exploring the subjective perspectives of this population. Despite a comprehensive search, only 10 studies were eligible for inclusion, highlighting the limited evidence base. Future work should examine friends, acquaintances and neighbours as a distinct subgroup of informal caregivers, clearly differentiated from family caregivers.

## Supplementary Information

Supplementary Material 1. Final search strategies

## Data Availability

The set of studies analysed within the scoping review is available from the corresponding author upon reasonable request.
